# Validation of Reference Genes for Oral Cancer Detection Panels in a Prospective Blinded Cohort

**DOI:** 10.1371/journal.pone.0158462

**Published:** 2016-07-13

**Authors:** Jack L. Martin

**Affiliations:** Sharpe Strumia Research Foundation, 130 South Bryn Mawr Avenue, Bryn Mawr, Pennsylvania, 19010, United States of America, and PeriRx LLC, 370 Reed Road, suite 200, Broomall, Pennsylvania, 19008, United States of America; University of Lleida, SPAIN

## Abstract

**Background:**

Reference genes are needed as internal controls to determine relative expression for clinical application of gene expression panels. Candidate constitutively expressed genes must be validated as suitable reference genes in each body fluid and disease entity. Prior studies have predominantly validated oral squamous cell carcinoma associated messenger RNAs (mRNAs) based on quantitative polymerase chain reaction (qPCR) quantification cycle (Cq) values without adjustment for housekeeping genes.

**Methods:**

One hundred sixty eight patients had saliva collected before clinically driven biopsy of oral lesions suspicious for cancer. Seven potential housekeeping mRNAs and six pre-specified oral cancer associated mRNAs were measured with qPCR by personnel blinded to tissue diagnosis. Housekeeping gene stability was determined with the NormFinder program in a training set of 12 randomly selected cancer and 24 control patients. Genes with stability indices <0.02 were then tested in the validation set consisting of the remaining cancer and control patients and were further validated by the geNorm program. Cancer gene delta Cqs were compared in case and control patients after subtracting the geometric mean of the reference gene raw Cqs.

**Results:**

B2M and UBC had stability indices >0.02 in the training set and were not further tested. MT-ATP6, RPL30, RPL37A, RPLP0 and RPS17 all had stability indices <0.02 in the training set and in the verification set. The geNorm M values were all ≤1.10. All six pre-specified cancer genes (IL8, IL1, SAT, OAZ1, DUSP1 and S100P) were up-regulated in cancer versus control patients with from nearly twofold to over threefold higher levels (p<0.01 for all based on delta Cq values).

**Conclusions:**

Five reference genes are validated for use in oral cancer salivary gene expression panels. Six pre-specified oral carcinoma associated genes are demonstrated to be highly significantly up-regulated in cancer patients based on delta Cq values. These cancer and reference genes are suitable for inclusion in gene expression panels for research and clinical applications.

**Trial Registration:**

ClinicalTrials.gov NCT01587573

## Introduction

Salivary contains a broad range of biomarkers and has gained increasing interest as a readily accessible body fluid for disease detection and surveillance. [1] The salivary transcriptome has been demonstrated to change in the presence of systemic malignancy with disease specific footprints. [2] A group of mRNAs discovered to be up-regulated in the presence of oral squamous cell cancer have been confirmed in multiethnic cohorts and independently validated by the National cancer Institute–Early Detection Research Network. [3–6] Prior studies predominantly compared qPCR raw cycle threshold (Cq) values in oral cancer and control subjects without adjustment for rigorously selected internal reference genes. [3,6,7]

For clinical applications reference genes are needed to serve as an internal control for gene expression assays. [8,9] Robust reference genes that are stable between samples are necessary to detect subtle changes in gene expression and to correct for variability between assays performed at different times. [9,10] Constitutively expressed potential reference genes must be validated for each specific disease entity and in the body fluid or tissue of interest. [10,11] For, example, analysis by our group of patient level data from prior studies has demonstrated that commonly used reference genes can often show differences in expression in the saliva of cancer and control patients. [12] The goal of the present study is to validate reference genes suitable for salivary gene expression assays in an intended use population of patients with lesions suspicious for the presence of oral squamous cell carcinoma. The validated reference genes are also used to compare relative expression of six pre-specified oral cancer associated genes in cancer and control patients.

A prospective specimen collection with retrospective blinded evaluation (PRoBE) study design is employed. This design eliminates confounding related to selection bias that is potentially present in case control trials. [13]

## Materials and Methods

### Patient selection

The study was approved by the Institutional Review Boards of Michigan State University, the University of Michigan and the St John Providence Health System in Detroit, Michigan. One hundred sixty eight patients were recruited through the Michigan State University Department of Surgery, the University of Michigan Department of Surgery, and the St John Providence Health System clinics. Patients provided written informed consent before entering the trial. In addition to multicenter participation, patients were also enrolled from the community care referral offices of these institutions to insure that the study population was representative of general practice and included a broad spectrum of oral pathology. Enrollment began in May, 2012 and completed by June, 2014. Inclusion criteria included age over 18 years and requirement for a clinically-driven biopsy of an oral lesion suspicious for cancer. Exclusion criteria included previously diagnosed cancer other than non-melanoma skin cancer in the last five years or oral cancer in the last two years. Patients with cancer diagnosed earlier than this could be included if they were free of known disease and not on current treatment for cancer. Also excluded were patients with prior history of hepatitis, human immunodeficiency virus infection, autoimmune disorders or current immunosuppressive therapy. Biopsy specimens were evaluated in the clinical pathology departments of the respective institutions by pathologists with no knowledge of the biomarker results.

### Saliva Collection

Saliva was collected as previously described prior to oral lesion biopsy and the determination of oral cancer or benign disease. [3] Saliva was processed by previously described methods to obtain supernatant and was treated with SUPERase-INTM RNase inhibitor (20 U/mL) (Life Technologies, www.lifetechnologies.com). [3] Samples were frozen at -80°C prior to RNA isolation.

### Laboratory analysis

Laboratory analysis was performed by operators who were blinded to tissue diagnosis. Before the analysis of the clinical trial samples the mRNA extraction, pre-amplification and qPCR methods were validated to exceed the performance requirements of the Clinical Laboratory Improvement Amendments (CLIA) using volunteer saliva samples. Analyte sensitivity and specificity were also validated to exceed CLIA standards prior to study specimen analysis.

RNA was isolated from 300μL saliva supernatant using the MagMax Viral RNA Isolation Kit (Life Technologies) adapted on the KingFisher Flex 96 system (Thermo Scientific, Waltham, MA).

Reverse transcription (RT) and pre-amplification were performed using the SuperScript® III RT-PCR System with Platinum® Taq DNA Polymerase (Life Technologies). Primer sequences for the six pre-specified cancer genes (IL8, IL1, SAT, OAZ1, DUSP1 and S100P) were described previously and contain 21 to 28 bases. (Lee et al. 2011) Reference gene (B2M, MT-ATP6, RPL30, RPL37A, RPL0, RPS17 and UBC) primers were obtained from the TaqMan®OpenArray® human endogenous control panel (Life Technologies).

Quantitative PCR was setup and performed in triplicate for each sample. Cq values for the six pre-specified OSCC mRNAs and seven potential reference mRNAs were measured simultaneously on the QuantStudioTM 12K Flex Real-Time PCR System (ThermoFisher Scientific, Waltham, MA) according to the manufacturer’s instructions. Details of the qPCR, RT, pre-amplification and RNA isolation protocols are included in a supplemental file (S1 Appendix).

### Statistical methods

The NormFinder program was applied to the training set and stability values were generated. [14] The median Cq values were calculated for the seven candidate reference genes and compared in cancer and control with the Mann Whitney test. Messenger RNAs that had stability values <0.02 and had a p value for the difference in cancer and control of >0.10 were then further analyzed in the validation set. Stability values at this level are very robust and the p value of >0.10 was chosen to minimize the chance that there was a significant trend for a difference between cancer and control in the training set. [9] The NormFinder program was then applied to the subset of genes tested in the validation cohort and to the geNorm program for additional validation. [15]

Delta Ct values for the pre-specified cancer genes were calculated by subtracting the geometric mean of the validated reference genes from the raw Cq value of each cancer gene. This allows for the determination of relative expression of the cancer genes in comparison with non-cancer genes in individual patients and groups. [8,9] Delta Cq. values were then compared in cancer and control by the Mann Whitney test.

## Results

Patient demographics are summarized in Table 1. Patients with cancer were significantly older. Cancer patients had numerically more males, smokers and current alcohol drinkers, but this did not reach statistical significance.

**Table 1:** Patient Demographics.

	Cancer (n = 24)	Benign (n = 144)	p value
Age (years)	64±13	55±15	<0.05
Males	68%	55%	ns
Caucasians	87%	85%	ns
Smoking history	71%	60%	ns
Current alcohol use	64%	46%	ns

B2M and UBC had stability indices >0.02 in the training set. The difference in median Cq values between cancer and control for B2M and UBC were 1.4 and 1.3 with p values ≤0.10. These genes were not further tested in the validation set. MT-ATP6, RPL30, RPL37A, RPLP0 and RPS17 had stability values <0.02 in the training set and p values for caner versus control >0.10. The difference in Cq values for cancer and control for these five genes in the training set

Stability values for ATP6, RPL30, RPL37A, RPLP0 and RPS17 remained <0.02 in the validation set. (Table 2) The geNorm M values were all ≤1.10 (MT-ATP6 1.10, RPL30 0.78, RPL37A 0.60, RPLP0 0.62and RPS17 0.60) The difference in Cq values for these 5 genes between cancer and control remained insignificant in the validation set and ranged from 0.1 to 0.7 Cqs.

**Table 2:** Reference Gene Stability Values in Validation Set.

Gene name	Stability value
MT-ATP6	0.015
RPL30	0.008
RPL37A	0.002
RPLP0	0.007
RPS17	0.003

The delta Cq values of all six pre-specified cancer genes are significantly lower in cancer patients compared with controls. These Cq values are consistent with a nearly two to more than threefold increase in mRNA levels in cancer patients. (Table 3)

**Table 3:** Delta Cq Values of Cancer Genes (median and interquartile [IQ] ranges).

	IL1β	IL8	OAZ1	SAT	DUSP1	S100P
Cancer	-4.28	-4.35	0.28	-3.02	-0.21	0.46
IQ Range	-0.77 to 0.94	-4.82 to 3.35	-4.94 to -3.29	-3.83 to -2.67	-0.77 to 0.94	-0.60 to 1.06
Control	-3.02	-3.15	1.18	-1.75	1.43	1.21
IQ Range	-3.72 to -2.09	-3.76 to -2.32	0.40 to 2.03	-2.63 to -1.02	0.59 to 2.28	0.35 to 2.00
p value	<0.001	<0.001	<0.001	0.002	<0.001	<0.001

## Discussion

The use of saliva as a diagnostic bio-fluid has been under investigation for over a decade. The salivary transcriptome and proteome have been extensively studied and contain a large alphabet of potentially informative biomarkers for disease detection and surveillance. [1] Salivary transcriptome markers for oral squamous cell carcinoma detection have been verified in multiethnic cohorts and have been independently validated by the National Cancer Institute–Early Detection Research Network. [6] These prior studies were predominantly based on raw Cq values from qPCR laboratory analysis. For clinical applications reference genes are necessary to determine relative gene expression and to insure comparable results between analyses on different days. [8,9] At least 2 reference genes that are stable in expression within and between groups are recommended to detect changes in expression of disease related mRNAs. [9,10]

The present study utilized a PRoBE design, were samples were collected in the intended use population before diagnostic tissue biopsy. Samples were processed by laboratory personnel blinded to tissue diagnosis. Candidate reference mRNAs were tested for stability by NormFinder analysis as well as for equivalent raw Cq values in cancer and control patients in a randomly selected cohort. Qualifying genes were then validated in a second random cohort with the NormFinder program and were further validated by also applying the geNorm program. This trial design is consistent with National Cancer Institute recommendations for rigorous biomarker development. [13]

Several programs are available to determine the stability of potential reference genes. The NormFinder program was employed for the present study, because it allows for inter and intra group comparisons. In this model-based approach gene expression is compared by analysis of variance. The stability value for each gene represents the variation in gene expression across samples in a group and between groups. [14] Thus it is well suited for applications such as comparison of gene expression in cancer and control patients. Further validation was accomplished with the geNorm program, which provides information on the average pairwise variation of a reference gene with all other candidate genes. The M values generated by the geNorm program are all well within the range consistent with stable gene expression. [15]

Five reference genes were identified as stable within and between cancer and control groups. The stability values of <0.02 for all of these reference genes are very robust. These genes are thus very suitable for incorporation into marker panels for investigational and clinical use in oral squamous cell carcinoma detection. Utilizing these genes to determine the delta Cq values of six pre-specified cancer associated genes demonstrates highly significant up-regulation of all genes in this PRoBE design trial. This finding further validates the discriminatory value of these squamous cell carcinoma associated genes. In addition the identification robust reference genes for this specific application is mandatory for clinical use.

Other salivary transcriptome panels are under active clinical investigation for other malignancies and other systemic diseases. [12, 16–19] The demonstration that there are suitable reference genes that are stable in saliva is of vital importance in the development of clinically relevant biomarker panels for these additional diseases. However, each reference gene needs to be rigorously tested in the specific disease entity of interest to insure that they are not affected by the underlying disease process.

## Conclusion

The identification of robust reference genes for the use in salivary oral squamous cell carcinoma marker panels is of vital importance for clinical applications. Oral cancer associated mRNAs previously identified and validated based on raw Cq values are highly significantly up-regulated when analyzed based on delta Cq values after adjusting for reference genes. These cancer and reference genes are suitable for inclusion in gene expression assays for research and clinical applications. Salivary gene expression assays also hold promise for the identification of a number of other systemic diseases and for long-term disease surveillance.

## Supporting Information

S1 Appendix Laboratory analysis methodology. (DOCX)

S1 Data File Patient level laboratory data. (XLTX)
