# Supplementary material for: Validation of Reference Genes for Oral Cancer Detection Panels in a Prospective Blinded Cohort
Source: PLoS One. 2016 Jul 13;11(7):e0158462. doi: 10.1371/journal.pone.0158462 (PMC4943624; doi:10.1371/journal.pone.0158462)
Supplement: S1 Appendix — (DOCX) [file pone.0158462.s001.docx]

S1 Appendix

RNA Isolation and DNase Treatment

Reagents:

MagMax Viral RNA Isolation Kit (Life Technologies AM1939)

TURBO DNA-free Kit (Life Technologies AM1907)

Isopropanol, 100% Ethanol, 100%

RNA Isolation Control 1X TE Buffer

Protocol:

1. Prepare Mixes 1 and 2 as shown in the tables below:  
2. Dilute 10μL of the RNA Isolation Control in 290μL 1X TE Buffer. Prepare all samples with mix 1: 300μL saliva and 680μL mix 1. Mix by votexing 15 seconds.
3. Add 20μL of mix 2. Mix by vortexing. Incubate for 4 minutes with occasional mixing.
4. Place sample on magnetic stand for 3 minutes or until beads are separated from supernatant.  Aspirate and discard supernatant.
5. Remove the sample from the magnet. Add 300μL of Wash Solution 1. Vortex at moderate  speed for 30 seconds.
6. Place tube on magnetic stand for 3-5 minutes or until beads are separated from supernatant.  Aspirate and discard supernatant.
7. Repeat step 5-6 again for a total of two (2) Wash Solution 1 washes.
8. Remove the sample from the magnet. Add 450μL of Wash Solution 2. Vortex at moderate  speed for 30 seconds.
9. Place tube on magnetic stand for 3-5 minutes or until beads are separated from supernatant.  Aspirate and discard supernatant.
10. Repeat step 8-9 again for a total of two (2) Wash Solution 2 washes.
11. Leave samples on magnet for 2 minutes to dry.
12. Add 50μL of elution buffer. Vortex beads in solution for 4 minutes.
13. Place the sample on the magnetic stand for 3-5 minutes or until the beads are separated from the mixture.
14. Prepare Mix 3 (20% overage):   
15. Mix 6μL DNase Treatment Solution with ~50μL eluted RNA.
16. Incubate samples on a heat block at 37°C for 30 minutes.
17. Add 5μL of DNase Inactivator. Vortex sample for 10 seconds.
18. Incubate samples at room temperature for 5 minutes.
19. Centrifuge at >10,000rpm for 2 minutes.
20. Pipet the clear supernatant and store at -80°C or proceed to next step.

Pre-Amplification Setup

Reagents:

DNA/RNase-free water SuperScript III Taq qRT-PCR Kit (Life Technologies #11732-020) 2μM primer mix Internal Control Reverse Transcription Positive Control (RT PC)

Protocol:

1. Prepare the RT-PCR master mix as described in Table 1.  Table 1.

2. Dispense 7μL of RT-PCR master mix into each corresponding sample well of the cold 96-well PCR plate.

3. Add 1μL nuclease-free water to RT PC well.

4.  Add 1μL Internal Control to all sample wells and RNA Isolation Control. Do not add to RT PC well.

5. Add 2μL of RNA elute to each of the corresponding wells in the PCR plate.

6. Add 2μL RT PC to respective well.

7. Heat seal the plate with pierceable foil. Spin the plate briefly in the benchtop plate spinner and place  back into the cold block.

8. Immediately place the plate into the thermocycler and start the "Preamp" program:

9. Upon completion of the RT-PCR preamplification, add 90μL nuclease-free water to each well to prepare 1:10 dilutions of each sample. Seal plate with self-adhesive foil plate seal.

10.  Proceed to OpenArray PCR setup or store samples at -20°C for future use.

Open Array Protocol

Reagents:

TaqMan OpenArray Master Mix (Life Technologies 4462164)

qPCR Positive Control (PC)

Nuclease-free water

Protocol:

1. Remove an aliquot(s) of OpenArray MasterMix from 4°C.

2.  Use a silver sharpie to mark plate octants on a 384-well plate.

3. Use a repeater pipette to dispense 2.5μL of OpenArray Mastermix per well of the 384-well plate.

4. Using a 12-channel multichannel pipette to transfer 2.5μL of sample DNA to respective wells of the  384-well plate. Pipette three (3) times to mix.

5. Immediately seal plate with self-adhesive foil plate seals and use a rubber plate sealer to ensure  each sample well has been thoroughly sealed. Use a thin sharpie to mark your score lines.

6. Centrifuge the sealed plate at 2,000xg for 2 minutes to remove bubbles in the samples.

7. Remove plate from the centrifuge and score the foil with an X- Acto knife. Place plate in the  "Sample" location of the AccuFill instrument.

8. Load respective OpenArray plate(s) per equipment manual.

9.  When all plates are loaded and sealed, place into plate carrier and run in QuantStudio  12K Flex using the "OA Gene Expression" protocol.
